# A Case of Syphilis Suspected Preoperatively as a Case of Tongue Cancer

**DOI:** 10.1155/2023/9469814

**Published:** 2023-11-02

**Authors:** Shigeru Kondo, Ryuhei Okada, Yosuke Ariizumi, Takashi Kurita, Hiroshi Shintaku, Takahiro Asakage

**Affiliations:** ^1^Department of Otorhinolaryngology, Tokyo Medical and Dental University, Tokyo, Japan; ^2^Department of Head and Neck Surgery, Tokyo Medical and Dental University, Tokyo, Japan; ^3^Department of Infectious Disease, Tokyo Medical and Dental University, Tokyo, Japan; ^4^Department of Pathology, Tokyo Medical and Dental University, Tokyo, Japan

## Abstract

Syphilis is a sexually transmitted disease caused by *Treponema pallidum* (TP). We report a case of syphilis that was initially suspected as tongue cancer. An 86-year-old man consulted a neighborhood clinic with an approximately one-month history of pain in the right tongue. The result of scraping cytology of the tongue performed at the clinic was classified as class V, squamous cell carcinoma, and the patient was referred to our hospital. Physical examination revealed a mass on the right side of the tongue and a firm cervical mass. Biopsy revealed no evidence of malignancy; however, the imaging findings led to the suspicion of tongue cancer and lymph node metastasis. The results of blood examination revealed that the patient had syphilis, but since the patient showed few other symptoms, we decided to treat the infection after the planned surgery. We performed right partial glossectomy and neck dissection; however, the postoperative histopathology revealed no evidence of malignancy but nonspecific inflammatory changes with TP spirochetes. The incidence of syphilis has increased dramatically around the world, including Japan, during the last 20 years, and it no longer remains a rare disease. Therefore, syphilis should be included in the differential diagnosis of oral or cervical masses.

## 1. Introduction

Syphilis is a sexually transmitted disease caused by *Treponema pallidum* (TP) that affects multiple organs (tongue, lymph nodes, vessels, etc.). The number of syphilis cases has been increasing in recent decades, particularly in developed countries, in males who engage in sex with other males (MSM) [1, 2]. In 2020, 133,945 syphilis cases of all stages were reported by the US Centers for Disease Control and Prevention (CDC), with cases in MSM accounting for 43% of the 41,655 cases of primary and secondary syphilis in the US [1]. The trend has been similar in Japan; although the annual number of syphilis cases reported remained under 1000 between 2000 and 2010, the disease incidence has increased so rapidly that the number of cases exceeded 10,000 in 2022 [3]. It is vital that the government address this resurgence of syphilis to stem further spread of the disease.

Due to the diverse symptoms of syphilis, it is often difficult to distinguish syphilis from some other diseases, including malignant tumors, so that syphilis had come to be called the “the great imitator.” We herein report a case of syphilis that was initially suspected as a case of tongue cancer with cervical lymph node metastasis and was treated by glossectomy and neck dissection, in which the postoperative histopathology revealed the diagnosis of syphilis.

## 2. Case Presentation

**Figure 1:**
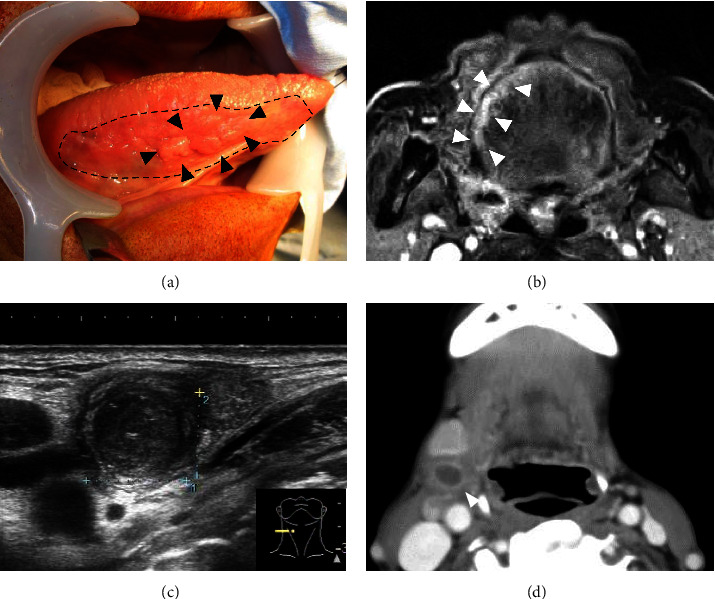
Clinical findings of the patient. (a) A mass lesion of the tongue. An exophytic mass measuring 20 mm in diameter was observed on the right side of the tongue (arrowheads), which was surrounded by a flat white leukoplakia-like lesion (dotted line). (b) Magnetic resonance imaging with gadolinium contrast. Enhancement of the lesion (white arrowheads) at the right edge of the tongue is observed. The tumor measured approximately 4 mm in thickness. (c) Ultrasound examination. An enlarged lymph node was detected in the superior internal jugular node area. The node was round and showed internal heterogeneous echoes; therefore, it was suspected as a metastasis. (d) Computed tomography with contrast. An enlarged lymph node is seen on the right side of the neck, containing a low-density area within and an enhancing edge, similar to the characteristic findings of lymph node metastasis from squamous cell carcinomas.

**Figure 2:**
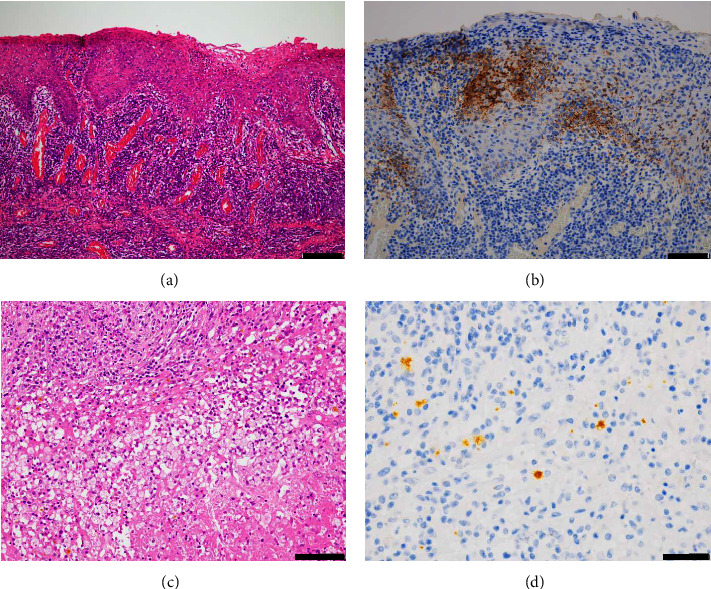
Pathological findings. (a) Hematoxylin and eosin staining of the tongue lesion. Reactive nuclear enlargement of the epithelial cells is seen, accompanied by neutrophilic infiltration of the epithelium and plasma cells into the subepithelial layer. No evidence of malignancy was detected. Scale bar = 200 *μ*m. (b) Immunohistochemical staining of the tongue lesion for *Treponema pallidum* (TP). Numerous spirochetes are seen within the epithelium. Scale bar = 100 *μ*m. (c) Hematoxylin and eosin staining of the suspected metastatic lymph node. Epithelioid cell granulomas with necrosis at the center of fibrotic changes are observed. Scale bar = 100 *μ*m. (d) Immunohistochemical stain of the lymph node for TP: multiple spirochetes are seen. Scale bar = 50 *μ*m.

An 86-year-old male patient visited a neighborhood clinic with an approximately one-month history of right tongue pain. The result of scraping cytology from the tongue performed at the clinic was categorized as class V, squamous cell carcinoma, and the patient was referred to our hospital for further treatment. Physical examination of the patient at our hospital revealed a mass lesion in the tongue measuring approximately 20 mm in diameter, with a 45-mm flat white leukoplakia-like lesion at the right edge of the tongue (Figure 1(a)); contrast-enhanced MRI showed enhancement of the tongue lesion, which measured 4 mm in thickness (Figure 1(b)). Palpation revealed an enlarged firm and relatively immobile lymph node of approximately 10 mm. Ultrasound examination showed a right cervical mass with internal heterogeneous echoes (Figure 1(c)). Contrast-enhanced CT revealed an enlarged right deep cervical lymph node with central necrosis (Figure 1(d)). We performed biopsy of the tongue tumor and a fine-needle aspiration biopsy of the enlarged cervical lymph node, but the results revealed no evidence of malignancy. At the same time, a preoperative routine blood examination revealed that the patient had syphilis infection; a rapid plasma reagin test revealed elevated antibody titer (1 : 1050), suggestive of a positive result for TP antibodies. Although the biopsy revealed no evidence of malignancy, we still strongly considered the possibility of tongue cancer with cervical lymph node metastasis, in view of the characteristics of the tongue lesion and imaging finding of the enlarged cervical lymph node with necrosis. Therefore, we proposed two treatment options to the patient and his family: (1) treatment of the syphilis infection, followed by watching the disease course, or (2) surgery for tongue cancer. The patient and his family chose (2). We performed right partial glossectomy and right neck dissection (levels I–V) with a plan to treat the syphilis after the surgery. Intraoperatively, we found that the enlarged lymph node that was suspected as a metastasis was severely adherent to the surrounding tissues. The findings of postoperative histopathology of the resected specimen are described in Figure 2. There was no evidence of malignancy either in the tongue or the lymph node. However, immunohistochemistry revealed spirochetes in the epithelium of the tongue as well as in the lymph node. The lymph node showed central necrosis, which led to the diagnosis of gumma. The biopsy specimen taken preoperatively was also retrospectively reevaluated with immunohistochemistry, which confirmed spirochetes infection.

After surgery, the patient was treated for syphilis with intramuscular benzylpenicillin potassium administered in three doses of 2,400,000 units each. He is currently under observation at our hospital. A careful review of his personal/social history revealed that the patient had engaged in vaginal intercourse with a suspected syphilis carrier at least a year before the appearance of his first symptoms.

## 3. Discussion

We report a case of syphilis that was initially suspected as tongue cancer. Historically, the inflammation induced by syphilis has been thought to be involved in the increased incidence of oral cancer [4, 5]. However, in a consensus report published by WHO in 2020, syphilis was excluded as an oral potentially malignant disorder (OPMD) [6]. It has also been reported that patients with both oral squamous cell carcinoma (OSCC) and syphilis did not have a poorer five-year survival probability as compared to patients with OSCC alone [7]. Therefore, syphilis is not regarded as a predisposing factor for tongue cancer at present.

Syphilis has diverse clinical manifestations, and clinicians often misdiagnose syphilis as other infections, malignant tumors, or autoimmune disorders, for which reason, the infection had come to be known as “the great imitator.” Depending on the duration of infection, syphilis is classified into four stages: primary, secondary, latent (hidden), and tertiary. The initial symptom pertains to the site of entry of the TP spirochetes. Painless lesions with ulcerative changes, known as “chancre,” are observed in the primary stage of syphilis, most frequently in the genital area and oral cavity. In regard with the primary and secondary stages of syphilis, the tongue, palate, and lips are reported as common sites for syphilitic lesions in the oral cavity [8]. Syphilis patients have also been reported to show enlarged lymph nodes with cystic or necrotic changes on CT images [9–11], very similar to the findings observed in cases of lymph node metastases from head and neck squamous cell carcinomas (HNSCCs), including tongue cancer.

There are no characteristic histopathologic findings of syphilis. Plasma cell infiltration is frequently observed in syphilis, but it is not a specific finding. In our case, spirochetes were observed in the gummatous lymph node with necrosis, despite their typical absence in such lesions. This unexpected finding explains the relatively early diagnosis of syphilis in our patient, as the spirochetes had not been completely eradicated by that stage. Moreover, cellular atypia in the initial scraping cytology could lead to the diagnosis of SCC. For patients with oral/oropharyngeal cancers, delayed diagnosis and treatment could result in an unfavorable prognosis of the patients [12–14] so that it is important for clinicians to make an accurate diagnosis as soon as possible. It is often difficult for clinicians to diagnose syphilis quickly and accurately even with the use of modalities like CT, MRI, biopsy, etc. In our case, we could not exclude tongue cancer from the findings of imaging examinations because the lymph nodes looked like metastatic lymph nodes on imaging examinations. However, clinicians should bear in mind the possibility of syphilis as an important differential diagnosis in patients presenting with oral or cervical masses, especially with the recent explosive increase in the incidence of syphilis.

## 4. Conclusion

We report a case of syphilis, in which an accurate diagnosis could be made only after surgery. It is often difficult for clinicians to correctly distinguish syphilis from other diseases (especially malignant tumors) due to the diverse clinical manifestations of syphilis. In addition to conducting a thorough physical examination and obtaining a detailed medical history, a key to the correct diagnosis is a careful review of the personal/social history of the patient. Considering the recent rise in the incidence of syphilis worldwide, it is important for clinicians to include this infection in the differential diagnosis of oral/cervical masses.

## Data Availability

The image data used to support the findings of this study are included within the article.
